# Supplementary material for: Iterative improvement in the automatic modular design of robot swarms
Source: PeerJ Comput Sci. 2020 Dec 7;6:e322. doi: 10.7717/peerj-cs.322 (PMC7924708; doi:10.7717/peerj-cs.322)
Supplement: Supplemental Information 3 [file peerj-cs-06-322-s003.zip › argos3/doc/api/standalone/a00502_source.html]

ARGoS: plugins/robots/generic/control\_interface/ci\_light\_sensor.cpp Source File


- Main Page
- Related Pages
- Namespaces
- Classes
- Files

- File List
- File Members

# plugins/robots/generic/control\_interface/ci\_light\_sensor.cpp

Go to the documentation of this file.

```
00001 
00007 #include "ci_light_sensor.h"
00008 
00009 #ifdef ARGOS_WITH_LUA
00010 #include <argos3/core/wrappers/lua/lua_utility.h>
00011 #endif
00012 
00013 namespace argos {
00014 
00015    /****************************************/
00016    /****************************************/
00017 
00018    const std::vector<Real>& CCI_LightSensor::GetReadings() const {
00019      return m_tReadings;
00020    }
00021 
00022    /****************************************/
00023    /****************************************/
00024 
00025 #ifdef ARGOS_WITH_LUA
00026    void CCI_LightSensor::CreateLuaState(lua_State* pt_lua_state) {
00027       CLuaUtility::StartTable(pt_lua_state, "light");
00028       for(size_t i = 0; i < m_tReadings.size(); ++i) {
00029          CLuaUtility::AddToTable(pt_lua_state, i+1, m_tReadings[i]);
00030       }
00031       CLuaUtility::EndTable(pt_lua_state);
00032    }
00033 #endif
00034 
00035    /****************************************/
00036    /****************************************/
00037 
00038 #ifdef ARGOS_WITH_LUA
00039    void CCI_LightSensor::ReadingsToLuaState(lua_State* pt_lua_state) {
00040       lua_getfield(pt_lua_state, -1, "light");
00041       for(size_t i = 0; i < m_tReadings.size(); ++i) {
00042          lua_pushnumber(pt_lua_state, i+1           );
00043          lua_pushnumber(pt_lua_state, m_tReadings[i]);
00044          lua_settable  (pt_lua_state, -3            );
00045       }
00046       lua_pop(pt_lua_state, 1);
00047    }
00048 #endif
00049 
00050 
00051    /****************************************/
00052    /****************************************/
00053 
00054 }
```

---

Generated on 10 Jul 2018 for ARGoS by 
 1.6.1
